# Evolution of therapeutic management of patients with ANCA associated vasculitis in France after licensing Rituximab use

**DOI:** 10.1186/s41927-024-00385-8

**Published:** 2024-04-28

**Authors:** Cécile-Audrey Durel, Eric Simon Thervet, Dominique Chauveau, Aurélie Schmidt, Benjamin Terrier, Pierre M Bataille

**Affiliations:** 1 Hôpital Saint Joseph Saint Luc, Lyon, France; 2 Hôpital Européen Georges Pompidou, Paris, France; 3 SORARE Centre Hospitalier Universitaire Rangueil, Toulouse, France; 4 Heva, Lyon, France; 5 Hôpital Cochin, Paris, France; 6 Centre Hospitalier Duchenne, Boulogne-sur-Mer, France

**Keywords:** Antineutrophil cytoplasmic antibody-associated vasculitides, Systemic vasculitis

## Abstract

**Introduction:**

In 2013, rituximab was approved in France for the treatment of ANCA-associated vasculitis (AAV). The aim of the study was to compare the treatment and health events of adult incident patients with granulomatosis with polyangiitis (GPA) and microscopic polyangiitis (MPA), included before rituximab approval (over 2010–2012, Group 1) and those included after rituximab approval (over 2014–2017, Group 2).

**Method:**

Data were extracted from the French National Health Insurance database (SNDS) including outpatient health care consumption and hospital discharge forms. Comparisons between inclusion periods were performed using Wilcoxon and χ² tests. Kaplan-Meier method was used to model the duration of treatment induction, maintenance, and off-drug periods. Fine and Gray tests were used to compare treatment phase durations.

**Results:**

A total of 694 GPA and 283 MPA patients were included in Group 1, while 668 GPA and 463 MPA patients were included in Group 2. Between the two inclusion periods, the proportions of patients treated with rituximab increased in the induction and maintenance phases whereas treatment with azathioprine declined. These proportions remained stable in the case of methotrexate, cyclophosphamide, and glucocorticoid-treated patients. Frequency of first-time hospitalized infections, diabetes and renal failure during the first year after inclusion increased for both groups.

**Limitations of the study:**

This is a retrospective study based on claims data including only 76% of people covered by health insurance in France. The period studied includes the learning phase of using rituximab. This study lacks biological data and precise quantitative analysis for the use of steroids, therefore the criteria for establishing diagnosis and therapeutic choice were unknown.

**Conclusions:**

Introduction of rituximab reduced the use of azathioprine without affecting the use of glucocorticoids or cyclophosphamide.

**Supplementary Information:**

The online version contains supplementary material available at 10.1186/s41927-024-00385-8.

## Key message

- Since its release in 2013, rituximab has been quickly adopted in current practice.
- Introduction of rituximab reduced the use of azathioprine but had minimal impact on glucocorticoid and cyclophosphamide use.
- A therapeutic management as effective as the current therapies and allowing to decrease the use of treatments with safety concerns such as glucocorticoid and cyclophosphamide is still needed.

**Supplementary Information:**

The online version contains supplementary material available at 10.1186/s41927-024-00385-8.

## Introduction

Granulomatosis with polyangiitis (GPA) and microscopic polyangiitis (MPA) are antineutrophil cytoplasmic antibody (ANCA)-associated vasculitides (AAVs). Both are rare diseases and, in France, in people 20 years old and above, the age-standardized prevalence rates are 10 and 4/100,000 person-years, respectively [1].

The therapeutic management of GPA/MPA patients has several goals, including to obtain disease remission and healing; to reduce the risk of relapse; and to limit the sequelae linked to the disease and its treatments [2]. The treatment encompasses two phases: an induction phase (lasting approximately 3–6 months) intending to obtain remission of the disease, followed by a maintenance phase (lasting 12 to 48 months according to history of relapse and ANCA type). There may be several induction treatments for disease flare-ups. Timely treatment initiation is key to prevent progression to kidney failure [3].

Since the 1970s, cyclophosphamide (CYC) in addition to corticosteroids are the backbone of the therapy for severe GPA and MPA. While therapies are often effective in inducing a remission, they are associated with a range of toxicities: for cyclophosphamide, increased risk of infections and cancer on the long term; for glucocorticoids (GCs), hypertension, osteoporosis, infections, and diabetes [4, 5]. Regimens associated to a reduced use of glucocorticoids and cyclophosphamide are therefore preferable to prevent these complications. In the 2010s, rituximab (RTX) was shown to be non-inferior to CYC in two clinical trials for AAVs and appeared to be more effective for relapsing disease in one clinical trial [6, 7]. Furthermore, RTX has a favourable safety profile [8] and proven efficacy [9]. The licensing of RTX for AAV in 2013 changed the patient management guidelines [10, 11] and was expected to lower GC use.

Drug use and safety in GPA and MPA in the real-world setting is yet to be quantified [12]. Hence, this observational study was set up in France with two goals. First, to describe the impact of the introduction of RTX on the therapeutic management of the diseases. Second, to describe in incident GPA/MPA patients the comorbidities and health events prior and within one year of their diagnosis.

## Materials and methods

### Study design and data sources

As described elsewhere [1], this retrospective longitudinal observational study was performed using data from the French National Health Data System (SNDS). Used for billing purposes, the SNDS contains demographic data and data on healthcare resource consumption in the community as well as in all hospitals and healthcare facilities [13]. The database does not provide the date of disease onset, but it can be approximated by the date of the first healthcare resource consumption (such as a hospital admission) for a disease.

### Study population and study period

All adult patients (≥ 18 years of age) with a unique social security number and hospitalized for GPA/MPA (identified with the principal diagnosis, associated diagnosis, or significant associated diagnosis in the hospital discharge form) between January 1, 2010, and December 31, 2017, were included in the study. The ICD-10 codes of interest were M31.3 “Wegener’s granulomatosis” for GPA, and M31.7 “Microscopic polyangiitis” for MPA. Patients also had to be affiliated to the General Scheme (which covers 76% of the people living in France [13]) between January 1, 2006, and December 31, 2017.

For patients identified as having both GPA and MPA (several hospitalizations with diagnoses of GPA and of MPA), the following rules were applied:

- Patients with less than six hospitalizations were not classified.
- For patients with at least six hospitalizations, if at least 60% of hospitalizations had one diagnostic code for one of the pathologies, the patient was reclassified as having this pathology.

Thus, some patients could not be classified as GPA only or MPA only. If reclassification was not possible, patients were kept for pooled analyses (GPA and MPA patients).

A follow-back period of four years for all patients (starting as early as January 1, 2006) allowed for the identification of comorbidities and potential information on GPA/MPA. The information sought out were any hospitalization for GPA/MPA, or long-term disease status (LTD, an administrative status allowing full reimbursement of healthcare expenses related to a chronic condition) for GPA/MPA, or a GPA/MPA-specific treatment. This evidence allowed to discriminate prevalent and incidence MPA/GPA cases. Incident patients were defined as patients without any GPA and/or MPA information during the four years prior to inclusion. Therefore, incident cases encompass both new patients and patients with a relapse occurring at least four years after the previous interaction with the healthcare system for those diseases. Prevalent patients were those with at least one interaction with the healthcare system during the four years prior to their inclusion in the study.

The index date (the inclusion date) was defined as the first date with GPA or MPA information in the SNDS during the inclusion period (hospitalization, LTD, or treatment). Patients were followed from inclusion until death, resignation from the general insurance scheme or a local mutualist section (together covering 87% of the French population), or until the end of the study (December 31, 2018), whichever occurred first. All study participants were followed-up for at least one year (or until death if it occurred sooner than a year after inclusion).

To examine the effect of the approval of RTX in 2013 on the clinical practice, patients were grouped according to their inclusion period (Group 1: before the introduction of RTX, inclusion in 2010–2012; Group 2: after the introduction of RTX, inclusion in 2014–2017).

### Outcomes

The outcomes were examined overall, by type of vasculitis, and by inclusion period.

Patient characteristics were described at the index date for incident patients (age, gender, and LTD status).

History of comorbidities were sought for the four years prior to the index date and within the first year after inclusion in incident patients without history of comorbidities. The comorbidities and health events were: cardiovascular diseases (i.e., stroke [including ischemic stroke, haemorrhagic stroke and transient ischemic attack], coronary disease, peripheral arterial disease), diabetes mellitus, renal failure, renal transplant, malignancies (all types), osteoporosis (includes hospital admissions for fractures), and pulmonary and urinary tract infections (hospital admission for). They were looked for in the SNDS with algorithms based on those published by the National Health Insurance or published in other studies [14]. These algorithms use hospital diagnoses, LTD status, specific treatments, and medical procedures (Supplement Table 1).

Over the whole study period, the therapeutic management of incident patients was described with the list of drugs delivered. The treatment was divided between induction, maintenance, and off-drug phases (Table 1). The definition of the treatment phases relied on the GC dose and on the other treatments. For lack of exact number of pills taken each day by each patient, this dose was defined as the number of tablets per box of medicine dispensed, multiplied by the number of boxes dispensed, spread over the 30 days following the dispensing date. A patient could have one or more induction and maintenance phases during the disease course.

**Table 1:** Definition of the induction phase, maintenance phase, off-drug phase, and refractory patients

	Induction phase	Maintenance phase	Off-drug phase	Refractory patients
Glucocorticoids (GC)	≥ 15 mg/day with or without a 30-day combination with MMF, AZA, or MTX	< 15 mg/day with or without a 30-day combination with MMF, AZA, or MTX	60 days without treatment or 180 days without treatment after 2 maintenance phases	
Mycophenolate mofetil (MMF)		MMF without a combined administration within 30 days of GCs		
Azathioprine (AZA)		AZA without a combined administration within 30 days of GCs		
Methotrexate (MTX)		MTX without a combined administration within 30 days of GCs		
Cyclophosphamide (CYC), intravenous (IV) or oral	CYC	No CYC		Switch from IV to oral *Or* Switch from CYC
Plasma exchange (PLEX)	PLEX	No PLEX		
Rituximab (RTX)	≥ 3 times over 3 months	< 3 times over 3 months		Switch from RTX
Infliximab (IFX)				IFX
Immunoglobulin (IG)				IG

The induction phase was defined as a delivery of glucocorticoids (GCs) with a dose ≥ 15 mg/day associated or not with a 30-day combination of mycophenolate mofetil [MMF], azathioprine (oral) [AZA] or methotrexate [MTX]), a delivery of CYC [intravenous or oral], a plasma exchange [PLEX] or a delivery of RTX with ≥ 3 administrations per three-month period. The maintenance phase was defined as an administration of GCs with a dose < 15 mg/day (with or without a 30-day combination of MMF, AZA or MTX); or an administration of MMF without a combined administration within 30 days of GCs; or an administration of AZA without a combined administration within 30 days of GCs; or a delivery of MTX without a combined administration within 30 days of GCs; or an administration of RTX with < 3 administrations per three- month period. If the patient received CYC or had a PLEX procedure, he/she was not in a maintenance phase. Off-drug phase was defined as period starting 60 days after the last treatment delivery, or starting 180 days after two maintenance phases back to back.

### Statistical methods

Continuous data were expressed as mean ± standard deviation (SD), median, and 1st and 3rd quartiles. Categorial data were expressed as numbers and percentages. Differences between GPA and MPA patients were tested with the Wilcoxon test (continuous variables such as the number of induction and maintenance phases) or Χ² (categorical variables such as the proportion of patients treaded with one drug). Kaplan-Meier method was used to model the duration of induction, maintenance, and off-drug phases. Fine and Gray tests were used to compare treatment durations between Group 1 and Group 2. All statistical tests were two-sided and were performed at a 5% significance level. Given that all healthcare consumptions are reported in the database, no replacement of missing values was performed.

Statistical analyses were performed using SAS version 9.4.

The STROBE guidelines were followed.

## Results

### Study patients

**Fig. 1:**
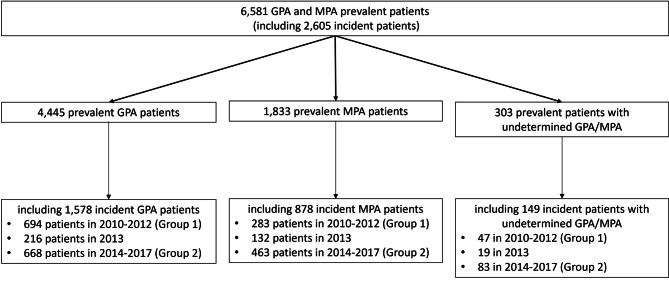
Study population

A total of 6,581 patients were enrolled over the study period, including 2,605 incident patients (39.6%) (Fig. 1). Amongst the incident patients, 1,578 patients suffered from GPA (60.6%) and 878 patients from MPA (33.7%). For 149 patients, it was not possible to classify them as GPA or MPA (5.7%). These patients with undetermined disease were pooled with GPA and MPA patients when presenting the results for all incident GPA/MPA patients.

Incident patients were divided in 2 groups based on their index date: 1,024 patients were included in Group 1(before 2013), and 1,214 patients in Group 2 (after 2013).

### Characteristics of incident patients at inclusion

The mean age of incident GPA and MPA patients was around 60.0 and 66.5 years old, respectively, and was stable during the two inclusion periods (Table 2).

The sex ratio was also stable over time: around 128 men for 100 women in GPA patients and around 105 men for 100 women in MPA patients.

In Group 1 and Group 2 GPA patients, 47% and 44% were hospitalized at least once in a centre of expertise during the study, respectively. In both Groups of MPA patients, 45% were hospitalized at least once in a centre of expertise.

More MPA (35.7% in Group 1 and 36.3% in Group 2) than GPA patients (18.7% in Group 1 and 15.4% in Group 2) suffered from renal failure at inclusion and over the four years prior to inclusion (*p* < 0.0001) (Supplement Fig. 1). Likewise, more MPA than GPA patients were hospitalized for pulmonary or urinary infection at the time of inclusion or during the prior four years (21.2% and 21.0% in MPA Groups 1 and 2, and 14.6% and 16.8% in GPA Groups 1 and 2, respectively) (*p* = 0.001).

**Table 2:** GPA and MPA incident patients’ characteristics at inclusion, by inclusion period

	GPA			MPA			GPA, MPA, and undetermined		
Inclusion period	2010–2012 *N* = 694	2014–2017 *N* = 668	P-value	2010–2012 *N* = 283	2014–2017 *N* = 463	P-value	2010–2012 *N* = 1,024	2014–2017 *N* = 1,214	P-value
Age (years), mean (± SD)	59.8 (± 15.5)	60.8 (± 15.7)	0.180	66.0 (± 13.4)	67.5 (± 13.2)	0.123	61.7 (± 15.3)	63.8 (± 15.1)	**0.001**
No. of men (%)	386 (55.6%)	376 (56.3%)	0.804	147 (51.9%)	247 (53.4%)	0.709	556 (54.3%)	664 (54.7%)	0.850

P-value in bold type means that the difference in the characteristics between the two inclusion periods is statistically significant

### Treatment phases in incident patients

#### Induction phases

The median number of induction phases in Group 1 GPA incident patients was 2. Group 2 GPA incident patients and Groups 1 and 2 MPA incident patients all had a median number of induction phases of 1(Table 3). The median phase duration was around 3 months in GPA patients, and decreased between the two inclusion periods from 3.4 to 2.9 months in MPA patients.

#### Maintenance phases

The median number of maintenance phases was 2 in both GPA and MPA incident patients and during both inclusion periods. In GPA patients, the median maintenance phase durations were 3 and 4.5 months in the two inclusion periods, respectively. In MPA patients, it increased from 3.7 to 5.4 months.

**Table 3:** Induction and maintenance phases in GPA and MPA incident patients, by inclusion period

	GPA		MPA		GPA, MPA, and undetermined	
Inclusion period	2010–2012	2014–2017	2010–2012	2014–2017	2010–2012	2014–2017
Induction phases in patients with at least one induction phase						
Number of patients	*N* = 610	*N* = 620	*N* = 251	*N* = 444	*N* = 903	*N* = 1,144
Median number of phases, (Q1–Q3)	2 (1–2)	1 (1–2)	1 (1–2)	1 (1–2)	1 (1–2)	1 (1–2)
Median phase duration (months)	2.9	3.1	3.4	2.9	3.1	3.0
Maintenance phases in patients with at least one maintenance phase						
Number of patients	*N* = 667	*N* = 635	*N* = 262	*N* = 433	*N* = 973	*N* = 1,145
Median number of phases, (Q1–Q3)	2 (1–3)	2 (1–3)	2 (1–2)	2 (1–3)	2 (1–3)	2 (1–3)
Median phase duration (months)	3.0	4.5	3.7	5.4	3.1	4.9

### Incident patients’ treatment distribution

#### Induction phase

Between those diagnosed in 2010–2012 and 2014–2017, the proportion of patients treated with RTX increased (from 8.4 to 24.6%) and with AZA decreased (from 29.6 to 14.0%) (Table 4). Despite the sharp increase in RTX use in Group 2, the percentage of patients treated with other drugs or with PLEX remained fairly stable.

In the case of GPA patients, a slight decrease in GCs treatment was also observed between the 2 periods (from 96.9 to 93.9%), while the proportion of patients treated by PLEX increased (from 17.7 to 23.1%). This was not observed for MPA patients. Other than for the higher use of MTX in GPA patients, treatment frequencies were similar between GPA and MPA groups.

The median GC durations were 6.5 and 7.6 months in GPA patients and 9.7 and 6.4 months in MPA patients (Supplement Table 2).

#### Maintenance phase

Between the two inclusion periods, the proportion of patients using RTX increased from 12.6 to 45.5% among GPA patients and from 9.9 to 40.9% among MPA patients. There was an important decline in AZA use in both GPA (from 38.4 to 20.9%) and MPA (from 40.1 to 28.6%) patients. The proportion of patients using GCs remained very high (at least 98% of patients) and stable over time, but the median GC treatment durations increased from 7.3 to 27.8 months in GPA patients and from 8.3 to 20.9 months in MPA patients between the two inclusion periods (Supplement Table 2).

**Table 4:** GPA and MPA incident patients’ treatments during the induction and maintenance phases, by inclusion period

	GPA			MPA			GPA, MPA, and undetermined		
Inclusion period	2010–2012	2014–2017	P-value	2010–2012	2014–2017	P-value	2010–2012	2014–2017	P-value
**Induction** phases in patients with at least one induction phase									
Number of patients	*N* = 610	*N* = 620		*N* = 251	*N* = 444		*N* = 903	*N* = 1,144	
Glucocorticoids	591 (96.9%)	582 (93.9%)	**0.012**	239 (95.2%)	425 (95.7%)	0.758	869 (96.2%)	1,084 (94.8%)	0.112
Cyclophosphamide	316 (51.8%)	289 (46.6%)	0.069	106 (42.2%)	207 (46.6%)	0.264	443 (49.1%)	523 (45.7%)	0.133
Rituximab	59 (9.7%)	171 (27.6%)	**< 0.001**	15 (6.0%)	89 (20.1%)	**< 0.001**	76 (8.4%)	281 (24.6%)	**< 0.001**
Azathioprine	178 (29.2%)	82 (13.2%)	**< 0.001**	72 (28.7%)	66 (14.9%)	**< 0.001**	267 (29.6%)	160 (14.0%)	**< 0.001**
Methotrexate	51 (8.4%)	65 (10.5%)	0.203	7 (2.8%)	10 (2.3%)	0.660	60 (6.6%)	79 (6.9%)	0.816
Mycophenolate mofetil	25 (4.1%)	25 (4.0%)	0.953	21 (8.4%)	22 (5.0%)	0.073	50 (5.5%)	49 (4.3%)	0.189
Plasma exchange	108 (17.7%)	143 (23.1%)	**0.020**	61 (24.3%)	99 (22.3%)	0.546	179 (19.8%)	261 (22.8%)	0.102
**Maintenance** phases in patients with at least one maintenance phase									
Number of patients	*N* = 667	*N* = 635		*N* = 262	*N* = 433		*N* = 973	*N* = 1,145	
Glucocorticoids	655 (98.2%)	625 (98.4%)	0.754	260 (99.2%)	423 (97.7%)	0.129	958 (98.5%)	1,123 (98.1%)	0.506
Rituximab	84 (12.6%)	289 (45.5%)	**< 0.001**	26 (9.9%)	177 (40.9%)	**< 0.001**	116 (11.9%)	498 (43.5%)	**< 0.001**
Azathioprine	256 (38.4%)	133 (20.9%)	**< 0.001**	105 (40.1%)	124 (28.6%)	**0.002**	380 (39.1%)	278 (24.3%)	**< 0.001**
Methotrexate	83 (12.4%)	75 (11.8%)	0.727	12 (4.6%)	13 (3.0%)	0.279	97 (10.0%)	92 (8.0%)	0.120
Mycophenolate mofetil	54 (8.1%)	31 (4.9%)	0.019	30 (11.5%)	42 (9.7%)	0.463	89 (9.2%)	78 (6.8%)	**0.047**

P-value in bold type means that the difference in the proportion of patients treated with one drug between the two inclusion periods is statistically significant

### Health events during the first year after inclusion

**Fig. 2:**
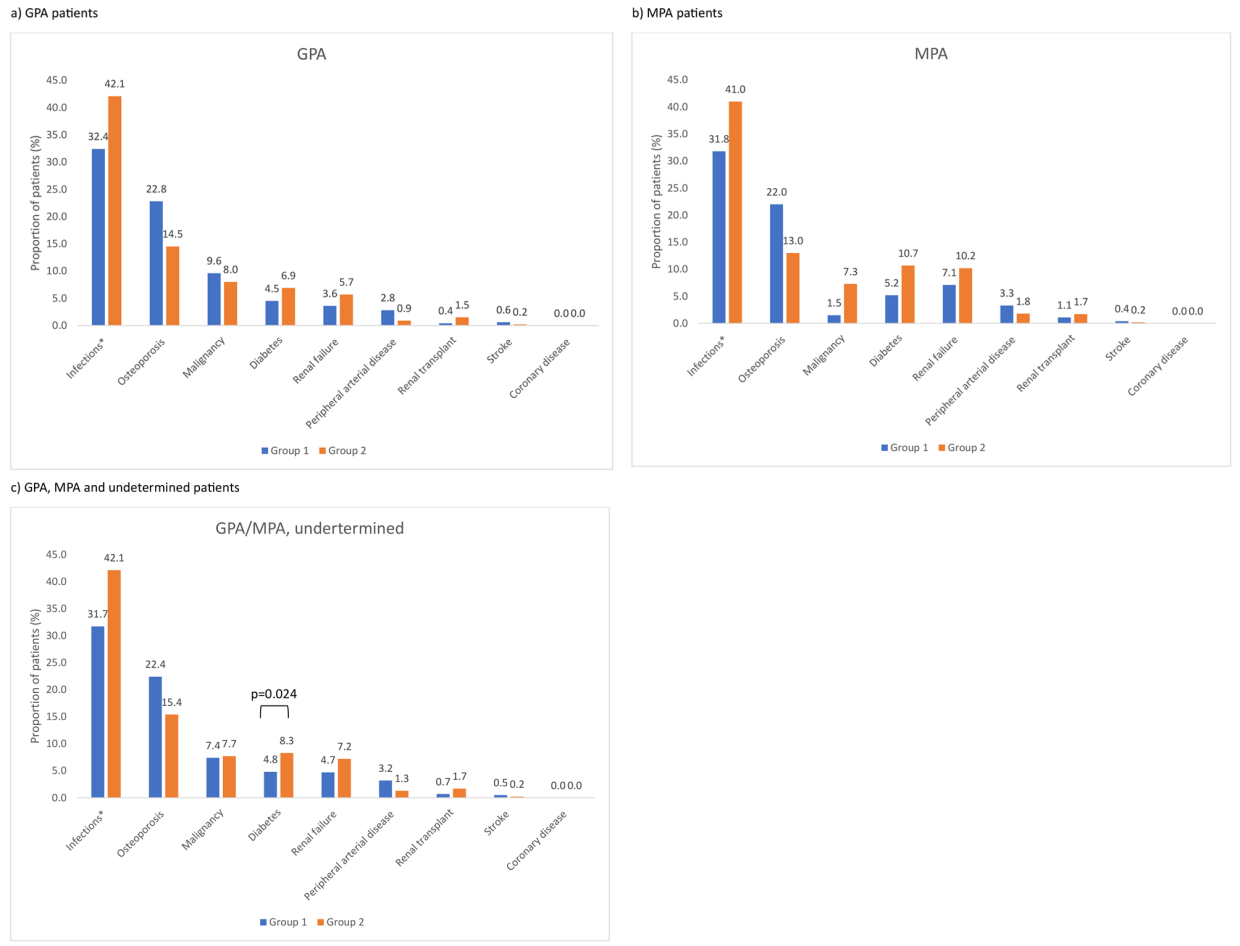
Health events within one year of the inclusion among patients without the health event at inclusion, in GPA and MPA incident patients. *Infections: pulmonary and urinary tact infections. Group 1: patients included in 2010-2012; Group 2: patients included in 2014-2017

Health events after inclusion were searched among patients with no history of the health event of interest. Within one year of inclusion, the most frequent health events were hospitalization for infection, osteoporosis, and malignancies (Fig. 2). The proportion of patients with infections or diabetes increased in both vasculitis types between the two inclusion periods; conversely it declined for osteoporosis. Regardless of the inclusion period, renal failure was more frequent in MPA than GPA patients.

## Discussion

Over 2010–2012 and 2014–2017, 1,024 and 1,214 incident GPA/MPA patients were included, respectively. Regardless of the inclusion period, the median number of the induction phases was one (except for the median number of induction phases in the first inclusion period of GPA patients which was two) and the median number of maintenance phases was two. As expected, the proportion of patients treated with RTX in the induction phase increased between those included before and after 2013 — RTX obtained the indication for the induction phase in France in 2013. However, an increase between the two inclusion periods was noted in the proportion of patients treated with RTX to maintain remission, while RTX did not have this indication yet, possibly following the positive outcome of the French MAINRITSAN (Maintenance of Remission using Rituximab in Systemic ANCA-associated Vasculitis) clinical trial [15]. Some of the practitioners of the centres of reference also participated in the clinical trial, hence it was straightforward for the practitioners to apply the findings of the trial. In parallel, the proportion of patients treated with AZA in the maintenance phase declined over time. Overall, the proportion of patients treated with MTX during the maintenance phase was stable between the two inclusion periods, probably because it was administered to less severe patients. The observed durations of the maintenance phases were shorter than expected, possibly because some maintenance phases may have been interrupted by the need for a new induction phase. Meanwhile, the observed duration of the GC maintenance therapy in the second inclusion period was consistent with what was expected, maybe because patients less often needed renewed induction phases. In general, GCs were as often used before and after 2013. One year after inclusion, the proportion of patients with first time hospitalized infections, diabetes or renal failure increased between 2010 and 2012 and 2014–2017.

The main challenge of this study has been the distinction between the induction and the maintenance phases, as there was no obvious cut-off between the two. Eventually, the most suitable definition of the two phases was based on the GC dose and on the other treatments. Nevertheless, the definition criteria did not fit all patient treatment protocols because more maintenance phases than induction phases were identified. Moreover, the median durations of the induction phase (close to 3 months) and the maintenance phase (about 3–5 months) were shorter than previously reported and recommended (3–6 months for the induction phase [16], 12–48 months or even longer for the maintenance phase [2]). In addition, anticipating earlier GC withdrawal, the maintenance phases’ durations were expected to be shorter after the launch of RTX. This was not observed in this study (only the median induction phase GCs duration in MPA patients declined). Regardless of the challenges of the phases’ definitions, RTX use increased over time and became part of clinical practice. The introduction of RTX only had a minimal impact on GC and CYC use (and PLEX in MPA patients).

Except for the duration of the maintenance treatment, we observed that the patient therapeutic management followed the current guidelines [2, 10] in two ways. First, due to the low prevalence of AAVs, it is recommended to manage patients in close collaboration with, or at, centres of expertise. Indeed, half of the patients were hospitalized at a centre of expertise at least once during the study. Yet the actual involvement of centres of expertise is likely underestimated in our study because patients are often referred to a centre of expertise for diagnosis confirmation, therapeutic management, complex cases, or complications but not always hospitalized. Second, the treatment of AAV should be based on GC and immunosuppressants and tailored to patients’ needs. Both GPA and MPA patients were treated with GCs and more than half of them had a treatment induction phase with GCs and either CYC or RTX.

Several health events were examined in our study during the year after inclusion. First, infections because they are common in patients with AAV [17]; pulmonary and urinary tract infections being among the most common ones [18–20]. During the one-year follow-up, 32% and 41% of patients included during the first and second inclusion periods, respectively, suffered from an infection, in accordance with other studies [21–23]. It was not possible to determine if the treatment changes between the two periods triggered higher infection rates. We have no specific biological data to assess the occurrence of hypogammglobulinemia linked to rituximab use. After 2014, the increase in the duration of induction treatment with prolonged use of steroids is a possible explanation for this increase. Then, osteoporosis was the second leading health event observed in our study. However, osteoporosis is more of a long-term adverse event [24] and its diagnosis is more likely to be due to screening for osteoporosis shortly after the AAV diagnosis than the development of osteoporosis within such short time frame. The decline in the proportion of patients newly diagnosed with osteoporosis in the second inclusion period could be due to the therapeutic prevention of treatment-related osteoporosis as stated in national guidelines [11]. Nevertheless, the proportion of patients diagnosed with osteoporosis was higher than in a previous Dutch study [25]. The decline in the frequency of osteoporosis in patients included in 2014–2017 could be due to a decrease in the cumulative dose of GC but the GC doses could not be quantified in a precise enough manner to enable us to test such hypothesis. On the contrary, renal failure has biological diagnoses and would not go unnoticed. Combining the proportion of patients with renal failure or transplantation, 8% of the study patients included in 2014–2017 suffered from severe nephropathy —an increase from those included in 2010–2012. This proportion seems underestimated compared to a retrospective observational study in Colombia in which 21% of patients had renal involvement of variable level, of which 54% progressed to advanced kidney disease [26]. The proportion of patients suffering from diabetes was close to that of a trial (7% at one year) [27]. The fact that the proportion of patients with diabetes increased in both vasculitis types between the two inclusion periods could be due to improved diagnosis. The proportion of patients treated with CYC remained stable before and after RTX authorization, as the proportion of patients diagnosed with cancer (7.4% and 7.7%, respectively). The use of CYC has been associated with an increased risk of urologic and hematologic malignancies [5] and a Dutch study concluded that the risk of cancer was lower among RTX-treated patients than among CYC-treated patients [28]. In the claims database we used, it is not possible to determine which factor(s) (a change in the therapeutic management or anything else) had a positive impact on the proportion new cancer cases.

### Strengths

The main strength of this study is to provide real-life data on the therapeutic management of AAV patients. Indeed, observational studies are fundamental to assess patients’ therapeutic management and health events because patients enrolled in clinical trials and included in observational studies have different characteristics [29]. Furthermore, it captures all treatments, as opposed to controlled trials [30]. This seven-year study allows to depict the therapeutic management of patients around the time of the groundbreaking authorization of RTX for AAV patients. The large study population allows to examine outcomes by vasculitides type and offers excellent representativity of the French population. The identification of patients is accurate because it is based on the hospital discharge database —all AAV patients are treated at the hospital. Finally, the retrospective data collection and the fact that data were collected for other purposes (healthcare reimbursement) guarantees the absence of patient selection bias.

### Limitations

Nevertheless, some limitations must be considered when interpreting the results. Classifying ANCA patients is challenging [31] and the absence of diagnoses and lab tests results prevented us from identifying the quality for the diagnosis process and vasculitis type. Namely in 6% of incident patients, we were unable to discriminate between GPA and MPA. Another main limitation was that the definition of induction vs. maintenance phase chiefly relies on the GC dosage, which is not readily accessible through the claims database. In addition, the claims database provides information of drug dispensing, not drug prescription nor drug taking. It is possible that some patients were not observant (as reported in long-term treatments [32]) but using claims data, it is impossible to know whether a patient is observant. As, by definition, treatment dosage and phase duration are linked in our study, it is also probable that, due to our definition of the maintenance phase dosage, treatment phases with low dosages were classified as multiple distinct treatment phases instead of a single one. Thus, this approximation of consumed doses to identify the induction and maintenance phases may have led to a classification bias. This bias is probably bi-directional, i.e., it is also possible that some treatment phases may have been wrongly classified as maintenance phases when they were not. Our definition of the maintenance phase of a GC dose of < 15 mg/day in combination with MMF, AZA, or MTX could potentially include patients with an induction therapy for a minor (non-major) manifestation. Taken together, these hypotheses could explain the unexpectedly short maintenance phase durations. Furthermore, the database does not contain information for the quality in therapeutical choice and roughly 50% of patients were not followed at a centre of expertise. Prescription and some treatments given in the management of GPA-MPA are not specific to those diseases. Particularly, GCs have multiple indications and could have been prescribed for a disease other than GPA/MPA. However, the tight selection of the study population based on reliable medical criteria ascertains that the patients included actually suffered from GPA/MPA, limiting the bias of these drugs being administered for a reason other than AAV. Reasons for discontinuation of treatment (off-drug periods) were not investigated. Our hypothesis is they could be due to treatment toxicity or disease-related events (osteoporosis, renal failure, malignancy, infections, etc.). Finally, the therapeutic management we observed is representative of 2010–2018. Since then, other treatments have been released, such as avacopan which could dramatically change how GPA/MPA patients are treated [33].

## Conclusion

This nationwide real-life study on GPA/MPA patients offers valuable insight on their therapeutic management and health events. The use of RTX in the induction phase met the guidelines and its use to maintain remission anticipated expert consensus guidelines. Even after the licensing of RTX, GCs remained a cornerstone of the therapeutic management of GPA/MPA patients despite known adverse events. Furthermore, RTX did not lead to a reduction in the use of CYC for the induction phase. As new therapies are authorized and patients’ survival increases over time, it is all the more important to prevent disease- and treatment-related comorbidities.

### Electronic supplementary material

Below is the link to the electronic supplementary material.

Supplementary Material 1

## Data Availability

The data underlying this article are part of the National health data system (SNDS, Système national des Données de Santé) and are available from the Health Data Hub (www.health-data-hub.fr). Restrictions apply to the availability of these data and they cannot be shared publicly. Permission to access data from the SNDS can be obtained by from the new the Ethics and scientific committee for health research, studies, and evaluations (CESREES, Comité Ethique et Scientifique pour les Recherches, les Etudes et les Evaluations dans le domaine de la Santé) (formerly CEREES) and the French data protection authority (Comité National de l’Informatique et des Libertés, CNIL).Consent for publication: Not applicable.

## References

[1] Bataille PM, Durel C-A, Chauveau D (2022). Epidemiology of granulomatosis with polyangiitis and microscopic polyangiitis in adults in France. J Autoimmun.

[2] Terrier B, Darbon R, Durel C-A (2020). French recommendations for the management of systemic necrotizing vasculitides (polyarteritis nodosa and ANCA-associated vasculitides). Orphanet J Rare Dis.

[3] Geetha D, Jefferson JA (2020). ANCA-Associated Vasculitis: Core Curriculum 2020. Am J Kidney Dis.

[4] Robson J, Doll H, Suppiah R (2015). Glucocorticoid treatment and damage in the anti-neutrophil cytoplasm antibody-associated vasculitides: long-term data from the European Vasculitis Study Group trials. Rheumatology.

[5] Koening CL, von Hennigs I (2021). Antineutrophil cytoplasmic antibody (ANCA) vasculitis: pathophysiology, diagnosis, and the evolving treatment landscape. Am J Manag Care.

[6] Stone JH, Merkel PA, Spiera R (2010). Rituximab versus Cyclophosphamide for ANCA-Associated Vasculitis. N Engl J Med.

[7] Jones RB, Cohen Tervaert JW, Hauser T (2010). Rituximab versus Cyclophosphamide in ANCA-Associated Renal Vasculitis. N Engl J Med.

[8] Merkel PA, Niles JL, Mertz LE (2021). Long-term safety of Rituximab in Granulomatosis with Polyangiitis and in microscopic polyangiitis. Arthritis Care Res.

[9] Charles P, Perrodeau É, Samson M (2020). Long-term Rituximab Use to maintain remission of Antineutrophil cytoplasmic antibody–Associated Vasculitis: a Randomized Trial. Ann Intern Med.

[10] Yates M, Watts RA, Bajema IM (2016). EULAR/ERA-EDTA recommendations for the management of ANCA-associated vasculitis. Ann Rheum Dis.

[11] Terrier B, Guillevin L. (2019) Protocole national de diagnostic et de soin des vascularites nécrosantes systémiques (périartérite noueuse et vascularites associées aux ANCA).

[12] Sultana J, Azzopardi-Muscat N, Coleiro B (2019). Pharmacological therapy in a rare disease: challenges in the long-term management of granulomatosis with polyangiitis. Expert Opin Orphan Drugs.

[13] Tuppin P, Rudant J, Constantinou P (2017). Value of a national administrative database to guide public decisions: from the système national d’information interrégimes de l’Assurance Maladie (SNIIRAM) to the système national des données de santé (SNDS) in France. Rev DÉpidémiologie Santé Publique.

[14] CNAM. (2021) Méthodologie médicale de la cartographie des pathologies et des dépenses, version G8 (années 2015 à 2019, Tous Régimes).

[15] Guillevin L, Pagnoux C, Karras A (2014). Rituximab versus Azathioprine for maintenance in ANCA-Associated Vasculitis. N Engl J Med.

[16] Samman KN, Ross C, Pagnoux C, Makhzoum J-P (2021). Update in the management of ANCA-Associated Vasculitis: recent developments and future perspectives. Int J Rheumatol.

[17] Thomas K, Argyriou E, Kapsala N (2021). Serious infections in ANCA-associated vasculitides in the biologic era: real-life data from a multicenter cohort of 162 patients. Arthritis Res Ther.

[18] Thomas K, Vassilopoulos D (2017). Infections and vasculitis. Curr Opin Rheumatol.

[19] Garcia-Vives E, Segarra-Medrano A, Martinez-Valle F (2020). Prevalence and risk factors for major infections in patients with Antineutrophil cytoplasmic antibody–associated Vasculitis: influence on the Disease Outcome. J Rheumatol.

[20] Caballero-Islas AE, Hoyo-Ulloa I, García-Castro A, Hinojosa-Azaola A (2020). Severe infections in patients with anti-neutrophil cytoplasmic antibody-associated vasculitis: a retrospective cohort study with a clinical phenotype approach. Rheumatol Int.

[21] Biedroń G, Włudarczyk A, Wawrzycka-Adamczyk K (2020). Treatment and its side effects in ANCA-associated vasculitides - study based on POLVAS registry data. Adv Med Sci.

[22] Walsh M, Merkel PA, Peh C-A (2020). Plasma exchange and glucocorticoids in severe ANCA-Associated Vasculitis. N Engl J Med.

[23] Sarica SH, Dhaun N, Sznajd J (2020). Characterizing infection in anti-neutrophil cytoplasmic antibody–associated vasculitis: results from a longitudinal, matched-cohort data linkage study. Rheumatology.

[24] Robson J, Doll H, Suppiah R (2015). Damage in the anca-associated vasculitides: long-term data from the European Vasculitis Study group (EUVAS) therapeutic trials. Ann Rheum Dis.

[25] Boomsma MM, Stegeman CA, Kramer AB (2002). Prevalence of reduced bone mineral density in patients with anti-neutrophil cytoplasmic antibody associated vasculitis and the role of immunosuppressive therapy: a cross-sectional study. Osteoporos Int J Establ Result Coop Eur Found Osteoporos Natl Osteoporos Found USA.

[26] Fernández-Ávila DG, Rondón-Carvajal J, Villota-Eraso C (2020). Demographic and clinical characteristics of patients with ANCA-positive vasculitis in a Colombian University Hospital over a 12-year period: 2005–2017. Rheumatol Int.

[27] Seo P, Min Y-I, Holbrook JT (2005). Damage caused by Wegener’s granulomatosis and its treatment: prospective data from the Wegener’s granulomatosis Etanercept Trial (WGET). Arthritis Rheum.

[28] van Daalen EE, Rizzo R, Kronbichler A (2017). Effect of rituximab on malignancy risk in patients with ANCA-associated vasculitis. Ann Rheum Dis.

[29] Pagnoux C, Carette S, Khalidi NA (2015). Comparability of patients with ANCA-associated vasculitis enrolled in clinical trials or in observational cohorts. Clin Exp Rheumatol.

[30] Charles P, Terrier B, Perrodeau É (2018). Comparison of individually tailored versus fixed-schedule rituximab regimen to maintain ANCA-associated vasculitis remission: results of a multicentre, randomised controlled, phase III trial (MAINRITSAN2). Ann Rheum Dis.

[31] Corral-Gudino L, González-Vázquez E, Calero-Paniagua I (2020). The complexity of classifying ANCA-associated small-vessel vasculitis in actual clinical practice: data from a multicenter retrospective survey. Rheumatol Int.

[32] Sabaté E, World Health Organization (2003). Adherence to long-term therapies: evidence for action.

[33] Pagnoux C, Fifi-Mah A (2021). Update on maintenance therapies for ANCA-Associated Vasculitis. Curr Treat Options Rheumatol.

