# Supplementary material for: Evolution of therapeutic management of patients with ANCA associated vasculitis in France after licensing Rituximab use
Source: BMC Rheumatol. 2024 Apr 28;8:16. doi: 10.1186/s41927-024-00385-8 (PMC11056056; doi:10.1186/s41927-024-00385-8)
Supplement: Supplementary file 1 — Supplementary Material 1 [file 41927_2024_385_MOESM1_ESM.docx]

Supplement

Evolution of the therapeutic management of patients with granulomatosis with polyangiitis and microscopic polyangiitis in the rituximab era: a nationwide observational study in France

Cecile-Audrey Durel, Éric Thervet, Dominique Chauveau, Aurélie Schmidt, Benjamin Terrier, Pierre M Bataille

[Supplement Table 1 Algorithms for identification of studied events 2](#_Toc125712994)

[Supplement Table 2 Median duration of the glucocorticoid treatment, by vasculitis type and by inclusion period 7](#_Toc125712995)

[Supplement Figure 1 Comorbidities four years before and at inclusion in incident, by vasculitis type and by inclusion period 8](#_Toc125712996)

# Supplement Table 1 Algorithms for identification of studied events

|  | Hospitalization | LTD | Procedures | Drugs |
| --- | --- | --- | --- | --- |
| Coronary diseases | At least one hospitalization for selected cardiovascular diseases (ICD-10 codes I20-I25) (PD/RD/SAD) during the year prior to index date | ICD-10 codes for selected cardiovascular events (I20-I25) during one year before index date |  |  |
| Peripheral arterial disease | At least one hospitalization for selected peripheral arterial diseases (ICD-10 codes I70, I702, I73, I739, I74, I740, I743, I744, I745) (PD/RD/SAD during the year prior to index date or PD/RD during the 4 years prior to index date) | ICD-10 codes for selected cardiovascular events (I20-I25) during one year before index date | At least one hospitalization for selected peripheral arterial procedures (CCAM codes DHCA004, EBAA002, EBAF001, EBAF003, EBAF004, EBAF005, EBAF006, EBAF009, EBAF010, EBAF011, EBCA001, EBCA002, EBCA004, EBCA008, EBCA010, EBCA011, EBCA013, EBCA015, EBCA017, EBEA003, EBEA005, EBKA001, EBKA002, EBKA003, EBKA004, ECFA003, ECFA004, ECLF004, EDCA004, EDCA005, EEAA002, EEAF001, EEAF002, EEAF003, EEAF004, EEAF005, EEAF006, EECA001, EECA002, EECA003, EECA005, EECA006, EECA007, EECA008, EECA010, EECA012, EEGA001, EEGA002, EEKA001, EELF002, EGAF003, EGCA003, EGFA006, EJCA002, EJCA003, EJEA001, EMSA001, ENAF001, ENAF002, ENFA005, EPCA002, EZCA002, EZCA003, EZCA004, EZFA003, EZPA001) during the 4 years prior to index date |  |
| Diabetes mellitus | At least one hospitalization for diabetes mellitus (ICD-10 codes E10-E14) (PD or RD) during the 2 years prior to index date and/ or at least one hospitalization for complication of diabetes mellitus (ICD-10 codes G59.0*, G63.2*, G73.0*, G99.0*, H28.0*, H36.0*, I79.2*, L97, M14.2*, M14.6*, N08.3*) (SAD / PD or RD of RUM) during the 2 years prior to index date (PD/RD) | ICD-10 codes for diabetes mellitus (ICD-10 codes E10-E14) during one year before index date |  | At least 3 dispensing (at different dates) of oral antidiabetic drugs or insulin (or 2 in case of at least one big box) the year prior to index date (ATC classes A10 excluding A10BX06) |
| Renal failure | At least one hospitalization for renal insufficiency, nephropathy, dialysis (PD/RD/SAD) during the 5 years prior to index date (ICD-10 codes I12, I13.1, I13.2, N18, Z49* or benefits 2129, 2131, 2132, 2134, 2135, 2136, 2137, 2138, 2139, 2140, 2142, 2143, 2144, 2145, 2146) | ICD-10 for renal failure, nephropathy, dialysis during the 5 years before index code (I12, I13.1, I13.2, N18, Z49*) |  |  |
| Renal transplantation | At least one hospitalization for renal transplantation (PD/RD/DRG during the 4 years prior to index date or PD/RD/SAD/DRG during the year prior to index date) (ICD-10 code Z94.0, DRGs 27C06Z, 27C061, 27C062, 27C063, 27C064, 24M39Z, 11M171, 11M172, 11M173, 11M174) |  | At least one hospitalization for renal transplantation procedure (JAEA003, HNEA002) |  |
| Malignancies (All types) | At least one hospitalization for malignancy (ICD-10 codes C00*-D09*, Z51.0, Z51.1) (PD/RD/SAD during the year prior to index date or PD/RD during the 4 years prior to index date) | LTD for malignancy during the year before index date (ICD-10 codes C00*-D09*) |  |  |
| Osteoporosis | At least one hospitalization for abnormalities of density and bone structure or vertebral fractures, fractures of upper extremity of femur and wrist (ICD-10 codes M80-M85, S62.0, S62.1, S52.5, S72.0) (PD/RD/SAD during the 4 years prior to index date or PD/RD during the year prior to index date) |  |  | At least one dispensing of a specific treatment for osteoporosis (bisphosphonates, SERM – selective oestrogen receptor modulators, strontium ranelate, denosumab, teriparatide) the year prior to the index date (ATC classes M05BA04, M05BA07, M05BA08, M05BB03, M05BX04, H05AA02, G03XC01, M05BB) |
| Pulmonary and urinary tract infections | At least one hospitalization for pulmonary or urinary tract infections (PD/RD during the 4 years prior to index date or PD/RD/SAD during the year prior to index date)  (ICD-10 codes A*, B* excluding B90* B91* B92* et B94*, H600, H601, H602, H603, H610, I301, I330, I400, J020, J028, J030, J038, J06*, J09*, J10*, J11*, J12*, J13*, J14*, J15*, J16*, J18*, J20*, J21*, J22*, J340, J36*, J390, J391, J440, J851, J852, J853, J86*, K046, K047, K113, K61*, K630, K650, K81*, L00*, L01*, L02*, L03*, L04*, L05*, L08*, L303, L305, L410, L411, L42*, L440, M00*, N10*, N11*, N12*, N136, N151, N340, N390, N412, M431, N450, N70*, N751, N764, O23*, O753, O85*, O86*, 091*, 098*, P35*, P36*, P37*, P38, P39*) |  |  |  |
| Stroke | At least one hospitalization for stroke (PD/RD during the 4 years prior to index date or PD/RD/SAD during the year prior to index date) (ICD-10 codes G45, I60-I64, I69) | LTD for stroke during one year before the index date (ICD-10 codes G45, I60-I64, I69) |  |  |

LTD: long-term disease; PD: principal diagnosis; RD: related diagnosis; SAD: significant associated diagnosis

Source: Caisse Nationale d’Assurance Maladie. Méthodologie médicale de la cartographie des pathologies et des dépenses, version G8 (années 2015 à 2019, Tous Régimes). 2021. <https://assurance-maladie.ameli.fr/content/methode-detaillee-du-reperage-de-56-pathologies-episodes-de-soins-ou-traitements>

# Supplement Table 2 Median duration of the glucocorticoid treatment, by vasculitis type and by inclusion period

|  | GPA | | MPA | | GPA, MPA, and undetermined | |
| --- | --- | --- | --- | --- | --- | --- |
| Inclusion period | 2010–2012 | 2014–2017 | 2010–2012 | 2014–2017 | 2010–2012 | 2014–2017 |
| Median induction phases duration (months) | 6.5 | 7.6 | 9.7 | 6.4 | 7.0 | 6.8 |
| Median maintenance phases duration (months) | 7.3 | 27.8 | 8.3 | 20.9 | 7.6 | 25.2 |

# Supplement Figure 1 Comorbidities four years before and at inclusion in incident, by vasculitis type and by inclusion period

## 1a) GPA patients

*Infections: hospitalization for pulmonary or urinary infection.

Group 1: patients included in 2010-2012; Group 2: patients included in 2014-2017

## 1b) MPA patients

*Infections: hospitalization for pulmonary or urinary infection.

Group 1: patients included in 2010-2012; Group 2: patients included in 2014-2017

## 1c) GPA, MPA, and undetermined patients

*Infections: hospitalization for pulmonary or urinary infection.

Group 1: patients included in 2010-2012; Group 2: patients included in 2014-2017
